# The cotton HD-Zip transcription factor GhHB12 regulates flowering time and plant architecture via the GhmiR157-GhSPL pathway

**DOI:** 10.1038/s42003-018-0234-0

**Published:** 2018-12-13

**Authors:** Xin He, Tianyi Wang, Zheng Xu, Nian Liu, Lichen Wang, Qin Hu, Xiangyin Luo, Xianlong Zhang, Longfu Zhu

**Affiliations:** 1 National Key Laboratory of Crop Genetic Improvement, Huazhong Agricultural University, 430070 Wuhan, Hubei China; 2 Southern Regional Collaborative Innovation Center for Grain and Oil Crops in China, Hunan Agricultural University, 410128 Changsha, Hunan China

**Keywords:** Agricultural genetics, Flowering, Patterning, Plant domestication

## Abstract

Domestication converts perennial and photoperiodic ancestral cotton to day-neutral cotton varieties, and the selection of short-season cotton varieties is one of the major objectives of cotton breeding. However, little is known about the mechanism of flowering time in cotton. Here, we report a cotton HD-ZIP I-class transcription factor (GhHB12) specifically expressed in axillary buds, which antagonisticlly interacts with GhSPL10/13 to repress the expression of *GhFT*, *GhFUL*, and *GhSOC1*, resulting in bushy architecture and delayed flowering under long-day conditions. We found that GhHB12-mediated ancestral upland cotton phenotypes (bushy architecture and delayed flowering) could be rescued under short-day conditions. We showed that overexpressing of *GhrSPL10* partially rescues the bushy architecture and delayed flowering phenotypes, while overexpression of *GhmiR157* reinforced these phenotypes in *GhHB12*-overexpressing plants. This study defines a regulatory module which regulates cotton architecture, phase transition and could be applied in the breeding of early maturing cotton varieties.

## Abstract (web summary)

Xin He et al. present a characterization of GhHB12, a HD-ZIP family transcription factor expressed in upland cotton axillary buds. They show that GhHB12 regulates flowering time, plant architecture and phase transition via a regulatory module that could be harnessed to improve cotton for mechanical harvesting.

## Introduction

Domestication is a selection process conducted by humans to adapt plants and animals to human needs. Despite the geographically diverse distribution of domestication centers, a remarkably similar set of traits (domestication syndrome traits) have been selected in a wide range of crops, including the architecture of plants, flowering time, distribution of reproductive structures, seed size, and dormancy^1^. The domestication of many crops resulted in more determinate architectures with compact growth habits, favorable and synchronous flowering times, and higher yields^2,3^. Over the past decade, researchers have identified some specific genes (domestication genes) that control the most important morphological changes associated with domestication.

Squamosa promoter-binding-like protein (SPL) is a plant-specific transcription factor family that regulates plant development and stress response, such as branch development, inflorescence development, timing of vegetative and reproductive phase change and response to stresses^4^. It is targeted by MicroRNA 156/157(miR156/157) for cleavage and/or translational repression, and both SPL and MicroRNA 156/157 are key genes in crop domestication^5,6^. In rice, OsSPL13 and OsSPL16 (SBP-Like) control grain size, shape, and quality via regulating cell proliferation^7,8^, and OsSPL14 controls panicle and shoot branching^9–11^. Moreover, novel functions were continuously recorded for SPL, such as the formation of the casing that surrounds the kernels of teosinte, fruit ripening of tomato, and tuberization of potato^12–14^. Overexpression of miR156/157 usually results in a bushy architecture and delays the flowering of plants via repressing SPL^7–11,15,16^. A common effect observed in domesticated crops is that the onset of flowering becomes less dependent on day length and vernalization, resulting in shorter life cycles, and the key domestication genes are the CENTRORADIALIS/TFL1/SP (CETS) family genes *FLOWERING LOCUS T* (*FT*) and *TERMINAL FLOWER1* (*TFL1*) and MADS-box genes *FLOWERING LOCUS C* (*FLC*), *SUPPRESSOR OF OVEREXPRESSION OF CONSTANS1* (*SOC1*), and *FRUITFULL* (*FUL*)^17–27^.

Upland cotton (*Gossypium hirsutum*) is the most important natural textile fiber source worldwide. The main stem of cotton is monopodial (meaning that the axis of growth continues from a single point) and remains indeterminate throughout development, producing lateral branches from the leaf axils indefinitely. Then, the lateral branches develop and differentiate into vegetative branches or fruiting branches. The vegetative branch often develops monopodially (meaning that the apical meristem is terminated and growth is continued by one or more lateral meristems, which repeat the process) from the lower leaf axils in the same way as the growth of the main stem, while the fruiting branch develops sympodially from the upper leaf axils, with each sympodial unit consisting of a terminal flower, a subtending leaf and an axillary bud to continue the branch^28^.

Ancestral upland cotton is mostly short-day photoperiodic, with exclusive monopodial (vegetative) branches and late-flowering under non-inductive long days. In contrast, domesticated cotton is day-neutral, with sympodial fruiting branches initiating as early as at the fifth to seventh node, and produces 1–3 vegetative branches below the node of the first fruiting branch (NFFB). Thus, fruit branching and NFFB are major events in the development of cotton architecture and important productivity-related agronomic traits considered in domestication programs^29,30^. Unfortunately, the regulatory mechanism of the transition from vegetative growth to reproductive development in cotton remains unclear.

Previously, an expressed sequence tag (EST), DW511649, was isolated as a candidate gene responsive to environmental stresses in cotton using data-mining and expression pattern analysis^31^. The full length of gene was obtained according to this EST, which putatively encodes a HD-ZIP I-class transcription factor, and was designated as *GhHB12*. Here, we demonstrate that this HD-ZIP transcription factor is specifically expressed in axillary buds, and can interact with GhSPL10 and GhSPL13. As a result, the expression of *GhFT*, *GhFUL*, and *GhSOC1* is repressed, which promotes cotton vegetative branch outgrowth and delays fruiting branch development under long-day conditions. In addition, the GhHB12-mediated ancestral upland cotton phenotypes (bushy architecture and delayed flowering) in cotton can be rescued under short-day conditions. Further analysis revealed that up-regulation of GhrSPL10 can also partially rescue the bushy architecture and delayed flowering phenotypes, while overexpression of *GhmiR157* reinforced these phenotypes in GhHB12-overexpressing plants. This study defines a novel regulatory module that regulates cotton architecture, phase transition and photoperiod sensitivity, which may help the breeding of early mature cotton varieties suitable for mechanical harvest.

## Results

### *GhHB12* promotes bushy architecture and delayed flowering

**Fig. 1:**
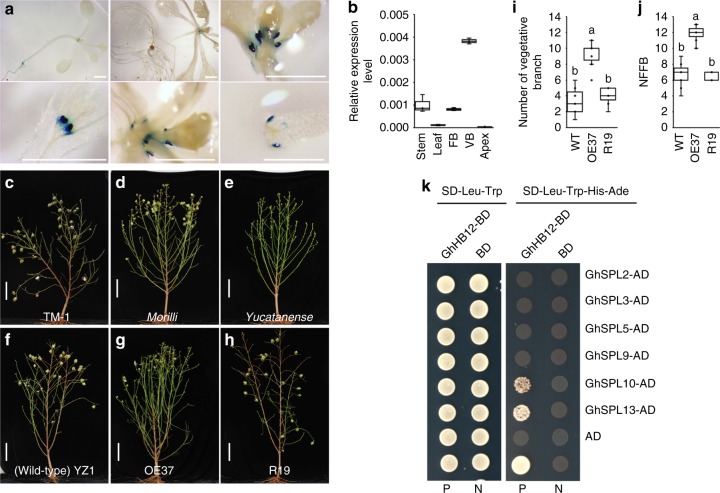
Identification of GhHB12. **a** Histochemical localization of GUS activity in *proGhHB12*:GUS *Arabidopsis* plants. Scale bars = 2 mm. **b** Detection of the expression of *GhHB12* in stem, leaf, fruit branch bud (FB), vegetative branch bud (VB), and apex of domesticated upland cotton (*Gossypium hirsutum* L. YZ1) by qRT-PCR. The *GhUBQ7* gene was used as the endogenous reference gene. The data represent the mean ± SD of three technical replicates. **c**–**h** Photographs of domesticated upland cotton TM-1 (**c**), YZ1 (**f**), OE37 (**g**, *GhHB12* overexpression in the YZ1 background), and R19 (**h**, *GhHB12* RNAi in the YZ1 background), ancestral upland cotton *morilli* (**d**) and *yucatanense* (**e**) plants. Scale bars = 20 cm. **i** and **j** Comparison of the number of vegetative branches (**i**) and the node of the first vegetative branch (NFFB) (**j**) among YZ1, OE37, and R19 plants. Error bars indicate the standard deviation of 12–15 plants, and different letters indicate significant differences at *P* < 0.05 (Duncan’s multiple range test). **k** Physical interaction of GhHB12 with GhSPL10 and GhSPL13 validated using the GAL-4-based Y2H assay. SD synthetic dropout, BD binding domain, AD activation domain, P positive control, N negative control

In our previous study, the cotton EST DW511649 was isolated as a putative abiotic-stress-responsive gene, which could be induced by salt and ABA treatments^31^. To further elucidate the function of DW511649, we cloned a putative full length cDNA sequence from domesticated upland cotton (*G. hirsutum*) variety YZ1 using rapid-amplification of cDNA ends (RACE) according to the sequence of DW511649. Alignment and phylogenetic analysis showed that this gene encoded a cotton HD-ZIP I-class transcription factor and had the highest similarity to *Arabidopsis* ATHB12, and thus was named as GhHB12 (Supplementary Figure 1a–1c). Spatial–temporal expression analysis showed that *GhHB12* was highly expressed in the buds of cotton vegetative branches (Fig. 1b). To further elucidate the expression profile of *GhHB12*, a 996-bp promoter region of *GhHB12* was isolated from YZ1, and several putative cis-acting regulatory elements involved in light-response, circadian control, stress-response and plant hormone-response were predicted (Supplementary Figure 1d and 1e), implying that *GhHB12* may function in the responses to various environmental cues. The 905-bp promoter region of *GhHB12* was fused to the GUS reporter gene and transformed into *Arabidopsis*. The *pro*GhHB12::GUS construct in transgenic *Arabidopsis* was predominantly expressed in axillary buds (Fig. 1a).

To evaluate the functions of GhHB12 in axillary bud and branch development, we constructed 35S::*GhHB12* and *GhHB12*-RNAi vectors, and *GhHB12*-overexpression (OE) and RNA interference (RNAi)-transformed plants were obtained with YZ1 through an *A. tumefaciens*-mediated method (see the experimental procedures). Three overexpressing lines (OE37, OE39, and OE42) and three RNAi lines (R7, R17, and R19) were selected for further analysis (Supplementary Figure 2a and 2b). Moreover, *GhHB12*-overexpressing *Arabidopsis* (monopodial growth pattern plant) and tomato (sympodial growth pattern plant) were obtained (Supplementary Figure 3). Among them, the *GhHB12*-overexpressing cotton lines showed phenotypes of more vegetative branches and delayed flowering time in Wuhan (China) under long day conditions. The growth of *GhHB12*-RNAi lines was similar to that of the wild type (YZ1), and the number of vegetative branches of *GhHB12*-RNAi lines was slightly smaller than that of the wild type (Fig. 1c–j and Supplementary Figure 2c–2k). Interestingly, the number of branches and flowering time showed no differences between *GhHB12*-overexpressing *Arabidopsis* lines and the wild type *Arabidopsis* (Col-0) (Supplementary Figure 3a), while overexpression of *GhHB12* in tomato led to late flowering and leafy inflorescences (Supplementary Figure 3b). These results indicated that GhHB12 is a crucial regulator of the sympodial growth pattern of plant branch development and phase transition.

### GhHB12 interacts with GhSPL10 and GhSPL13

SPL is a transcription factor family that regulates plant development, such as branch development, inflorescence development, and phase transition, and is regulated by MicroRNA 156/157(miR156/157)^16^. Overexpression of miR156/157 usually results in bushy architecture and delays flowering in plants^7–11,15,16^. The phenotypes of bushy architecture and inhibited flowering in *GhHB12*-overexpressing cotton plants led to the hypothesis that GhHB12 may interact with GhSPL. To test this hypothesis, we cloned six cotton miR156/157-targeted *GhSPLs* (*GhSPL2*, *GhSPL3*, *GhSPL5*, *GhSPL9*, *GhSPL10*, and *GhSPL13*), and detected the interactions between them and GhHB12 by the yeast two-hybrid system (Y2H) assay. As shown in Fig. 1k, the strains co-expressing GhHB12-BD (BD: GAL4 DNA-binding domain) and GhSPL10/13-AD (AD: activation domain) were able to grow on SD-Trp-Leu-His-Ade (medium lacking tryptophan, leucine, histidine, and adenine), whereas the clones expressing GhHB12-BD and GhSPL2/3/5/9-AD only grew on medium supplemented with histidine and adenine, indicating that GhHB12 interact with GhSPL10 and GhSPL13, but not with GhSPL2, GhSPL3, GhSPL5, or GhSPL9.

### GhHB12 and GhmiR157 coordinate in vegetative development

**Fig. 2:**
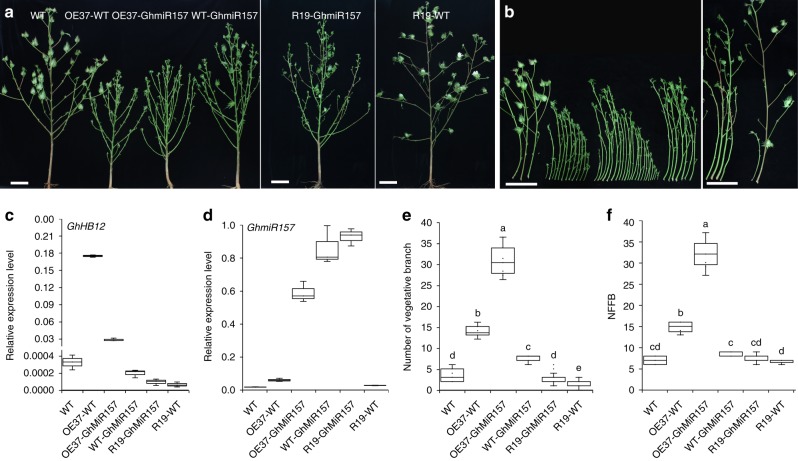
GhHB12 and GhmiR157 synergistically promote cotton vegetative branches and delay reproductive development. **a**–**f** Photographs of architectures (**a**) and vegetative branches (**b**), qRT-PCR analysis of the transcript levels of *GhHB12* (**c**), and *GhmiR157* (**d**), number of vegetative branches (**e**) and NFFB (**f**) of WT (wild-type), OE37-WT (hybrid plants of *GhHB12*-overexpression line and WT), OE37-GhmiR157 (hybrid plants of *GhHB12*-overexpression and *GhmiR157*-overexpression lines), WT-GhmiR157 (hybrid plants of WT and *GhmiR157*-overexpression line), R19-GhmiR157 (hybrid plants of *GhHB12*-RNAi and *GhmiR157*-overexpression lines), and R19-WT (hybrid plants of *GhHB12*-RNAi line and WT). The *GhUBQ7* gene was used as the endogenous reference gene. The data represent the mean ± SD of three technical replicates in **c** and **d**. Error bars indicate the standard deviation of 7–17 plants, and different letters indicate significant differences at *P* < 0.05 (Duncan’s multiple range test) in **e** and **f**

To further dissect the functions of cotton miR156/157 and SPLs, *GhmiR157*, *MIMI157* (GhmiR157 target mimicry vector generated by replacing the miR399 complementary motif of the 522-bp genomic sequence of *Arabidopsis* IPS1 with cotton *GhmiR157* complementary motif through PCR according to the previous study^32^), and *GhrSPL10* (a GhmiR157-resistant version without the GhmiR157 response element generated by introducing seven synonymous codon mutations into the predicted GhmiR157-binding site of GhSPL10 using recombinant PCR) were overexpressed in cotton as previously described^33–35^ (Supplementary Figure 4). Overexpression of *GhmiR157* greatly increased the number of vegetative branches^34^ and slightly delayed the flowering time in cotton (Fig. 2a and Supplementary Figure 4b). The growth of the *MIMI157*-overexpressing cotton line (designated as MIMI157-8) and *GhrSPL10*-overexpressing cotton line (designated as GhrSPL10-7) was similar to that of the wild-type YZ1 (Supplementary Figure 4c and 4d).

To investigate the possible genetic interactions between GhHB12 and GhmiR157, WT (wild-type YZ1) and GhmiR157-38 (*GhmiR157*-overexpressing cotton line) were crossed with the OE37 (*GhHB12*-overexpression line) and R19 (*GhHB12*-RNAi line), respectively, and the branching phenotype of the resulting F1 generation hybrid plants was examined. The vegetative branch number and NFFB of hybrid plants of *GhHB12*-overexpression and *GhmiR157*-overexpression lines were dramatically increased compared with those of hybrid plants of *GhHB12*-overexpression line and WT, and hybrid plants of *GhmiR157*-overexpression line and WT, whereas the vegetative branch number of hybrid plants of *GhHB12*-RNAi and *GhmiR157*-overexpression lines was significantly smaller than that of hybrid plants of *GhmiR157*-overexpression line and WT (Fig. 2), suggesting that GhHB12 and GhmiR157 synergistically promote the outgrowth of cotton vegetative branches and delay reproductive development.

**Fig. 3:**
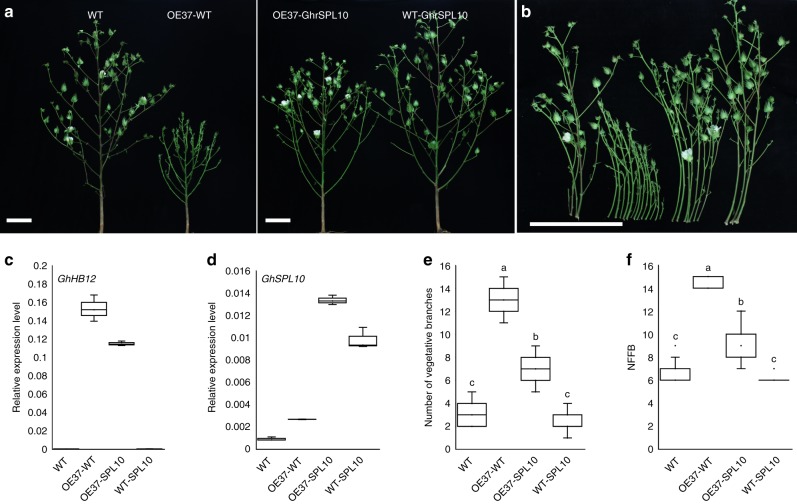
GhHB12 and GhSPL10 regulate vegetative branches and reproductive development in an antagonistic manner. **a**–**f** Photographs of architectures (**a**) and vegetative branches (**b**), qRT-PCR analysis of the transcript levels of *GhHB12* (**c**), and *GhSPL10* (**d**), number of vegetative branches (**e**) and NFFB (**f**) of WT (wild-type), OE37-WT (hybrid plants of *GhHB12*-overexpression line and WT), OE37-GhrSPL10 (hybrid plants of *GhHB12*-overexpression and *GhrSPL10*-overexpression lines), and WT-GhrSPL10 (hybrid plants of WT and *GhrSPL10*-overexpression line). The *GhUBQ7* gene was used as the endogenous reference gene. The data represent the mean ± SD of three technical replicates in **c** and **d**. Error bars in **e** and **f** indicate the standard deviation of 9–17 plants, and different letters indicate significant differences at *P* < 0.05 (Duncan’s multiple range test)

To further investigate the genetic interactions between *GhHB12* and *GhSPL10*, WT and GhrSPL10-7 plants were crossed with the OE37 and R19 plants, respectively, and the branching phenotype of the resulting F1 generation hybrid plants was examined. The vegetative branch number and NFFB of hybrid plants of *GhHB12*-overexpression and *GhrSPL10*-overexpression lines were significantly fewer than those of hybrid plants of *GhHB12*-overexpression line and WT (Fig. 3), suggesting that GhHB12 regulates cotton vegetative branch development and flowering time through antagonistically interacting with GhSPL10. Taken together, the results suggested that GhHB12 and GhmiR157-GhSPLs coordinate to regulate cotton architecture and phase transition.

### GhHB12 inhibits the expression of genes in phase transition

**Fig. 4:**
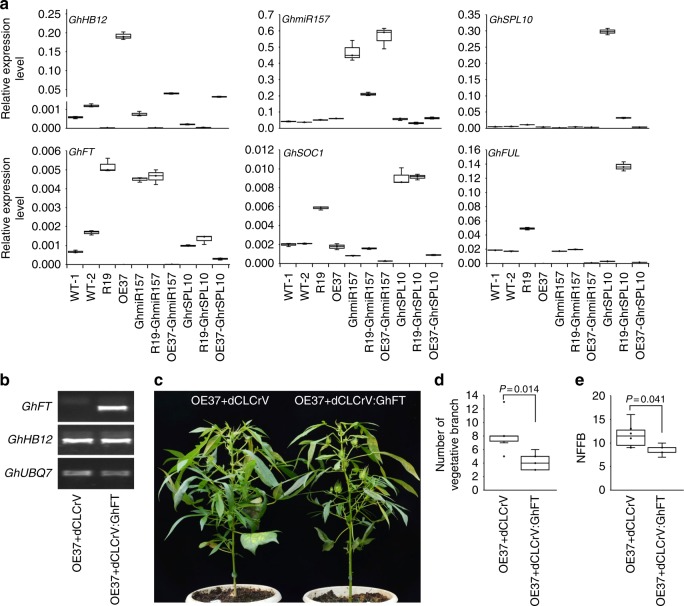
GhHB12 regulates cotton vegetative branches and reproductive development by suppressing *GhFT* and *GhFUL* transcription. **a** qRT-PCR analysis of the transcript levels of *GhHB12*, *GhmiR157*, *GhSPL10*, *GhFT*, *GhSOC1*, and *GhFUL* in WT and transgenic plants. The *GhUBQ7* gene was used as the endogenous reference gene. The data represent the mean ± SD of three technical replicates. **b** RT-PCR analysis of the transcript levels of *GhHB12* and *GhFT* in *GhHB12*-overexpressing plants inoculated with dCLCrV or dCLCrV:GhFT. **c** Photographs of GhHB12-overexpressing plants inoculated with dCLCrV or dCLCrV:GhFT. Number of vegetative branches (**d**) and NFFB (**e**) of *GhHB12*-overexpressing plants inoculated with dCLCrV or dCLCrV:GhFT. Error bars in **d** and **e** indicate the standard deviation of 5–6 plants. *P < 0.05 indicate significant differences between two groups (Student’s *t*-test)

It has been reported that some MADS transcription factors are the direct target genes of SPLs, such as *SOC1*, *FUL*, and *AP1*, which play important roles in plant phase transition and floral development^16,36^. To explore the downstream genes regulated by the GhHB12 and GhmiR157-GhSPLs pathways in cotton branch development and phase transition, we examined the expression levels of genes homologous to *SOC1*, *FUL*, and *AP1* in the leaves and shoot apices of five-leaf stage cotton seedlings. The results showed that *GhFUL* was dramatically repressed in *GhHB12*-overexpressing plants; *GhSOC1* was down-regulated in *GhmiR157*-overexpressing plants and was further repressed in OE37-GhmiR157 plants, whereas *GhSOC1*, *GhFUL*, and *GhAP1* were up-regulated in hybrid plants of GhHB12-RNAi and GhrSPL10-overexpression lines compared with in R19 and GhrSPL10 plants (Fig. 4a and Supplementary Figure 5a). In addition, the expression levels of some cotton *GhSPLs* decreased in GhmiR157-overexpressing plants and *GhHB12-*overexpressing plants, but were up-regulated in *GhHB12*-RNAi plants (Supplementary Figure 5a). These results demonstrated that GhHB12 suppresses the genes related to flowering time partly through the GhmiR157-GhSPLs pathway.

### GhHB12-mediated phenotypes are partially dependent on GhFT

CENRORADIALIS/TFL1/SP (CETS) family gene FLOWERING LOCUS T (FT) and its antagonist TERMINAL FLOWER1 (TFL1) play important roles in determinate growth and indeterminate growth of barley, sunflower, tomato, and strawberry^17,19,20,24,26,37^, and *FT* is directly regulated by SPL3 in *Arabidopsis*^38^. The variations of *GhFT* and *GhTFL1* activities would affect cotton architecture and photoperiod sensitivity. Overexpressing *GhSFT*/*GhFT* (*Gh_D08G2407* and *Gh_A08G2015*) in ancestral and photoperiodic cotton resulted in flowering under non-inductive long days, and silencing *GhFT* in domesticated and day-neutral cotton led to the phenotypes of more vegetative branches and delayed flowering time^21^. Therefore, we determined the expression of *GhFT* in the leaves and shoot apex of five-leaf stage seedlings and found that its expression was dramatically suppressed in *GhHB12*-overexpressing plants, while overexpression of *GhrSPL10* partially attenuated this suppression (Fig. 4a), implying that GhHB12 and GhSPL10 antagonistically regulate *GhFT*. Additionally, there was an inversely proportional relationship in the expression levels between *GhHB12* and *GhFT* in cotton vegetative branches and fruit branches (Fig. 1b and Supplementary Figure 5b), and the expression of either *GhHB12* or *GhFT* was rhythmic and regulated by photoperiod (Supplementary Figure 5c), which was consistent with the enrichment of light responsive cis-acting elements in the promoter of *GhHB12* (Supplementary Figure 1d). These results implied that GhHB12 represses the expression of *GhFT*, which may subsequently change the photoperiod sensitivity and architecture of cotton.

Interestingly, the phenotypes of bushy architecture and delayed flowering in *GhHB12*-overexpressing plants were completely rescued when grown in the Hainan (China) winter nursery (natural short-day). The growth of GhHB12-overexpressing plants was similar to that of the wild-type (YZ1) (Supplementary Figure 5d–5f). These results indicated that the photoperiodic effects of GhHB12 on the phenotype may depend on the expression of *GhFT*. To confirm this hypothesis, we transiently expressed *GhFT* in *GhHB12*-overexpressing plants via the dCLCrV system, a disarmed geminivirus *Cotton leaf crumple virus* was used for virus-induced flowering^21^. *GhHB12*-overexpressing plants inoculated with *dCLCrV:GhFT* flowered earlier than dCLCrV-infected *GhHB12*-overexpressing plants (Fig. 4b–e). Taken together, these results demonstrated that the regulatory effect of GhHB12 on cotton architecture, reproductive development, and photoperiod sensitivity is partially dependent on GhFT.

## Discussion

The main stem of cotton is monopodial and remains indeterminate throughout development, producing lateral branches (monopodial vegetative branches or sympodial fruiting branches) from the leaf axils indefinitely^28^. Cotton domestication converts perennial and photoperiodic ancestral cotton to day-neutral cotton varieties, and the selection of short-season cotton varieties suitable for mechanical harvest is a major objective of cotton breeding^39^. Although numerous QTLs regulating cotton branch development and flowering time have been identified^40–44^, little is known about the regulatory mechanism of cotton branch development and transition from vegetative growth to reproductive development.

Genes involved in axillary bud initiation control both vegetative and reproductive branching, whereas genes controlling axillary bud outgrowth have specific roles only at certain stages. Mir156/157-SPL plays vital roles in vegetative and reproductive phase changes, and overexpression of *miR156/157* usually results in a bushy and dwarf architecture and delays flowering in plants^4–6^. Similar to miR156/157, GhmiR157 could increase the outgrowth of vegetative branches in cotton. However, overexpression of *GhmiR157* slightly delayed the flowering time and increased plant height in cotton^34^ (Fig. 2 and Supplementary Figure 4b). These results demonstrate that the homologous genes and pathways among different species may have functional variations, and the branch development, phase transition, and plant height are controlled by a complex regulatory network in cotton.

In this study, we confirmed that a cotton HD-zip I class transcription factor GhHB12, which is specifically expressed in axillary buds (Fig. 1a, b) and directly interacts with miR156/157-targeted GhSPL10 and GhSPL13 (Fig. 1k), coordinates with the GhMir157-GhSPLs pathway to regulate the expression of genes related to cotton flowering time (e.g. *GhFT*, *GhSOC1*, and *GhFUL*), and subsequently modulates cotton architecture and phase transition (Figs. 2–4 and Supplementary Figure 5). It is noteworthy that overexpressing *GhHB12* in tomato (sympodial growth pattern plant) led to late flowering and leafy inflorescences, while it showed no effects on *Arabidopsis* (monopodial growth pattern plant) (Supplementary Figure 3). These results indicate that the monopodial branch and sympodial branch developments of different species are controlled in various ways, and GhHB12 is a crucial suppressor of the sympodial growth pattern of plant branch development and phase transition.

It has been reported that genes related to plant flowering time (*SOC1*, *FUL*, and *FT*) and the *SPL* genes could regulate each other in response to photoperiod signals to promote flowering^16,36,38,45^. *soc1* and *ful* mutants showed delayed flowering time in *Arabidopsis*^46,47^. Overexpression of *GhSOC1* promoted flowering in *Arabidopsis* and caused dwarfing of plant height in cotton^48^. *ft* mutant showed delayed flowering time in many plants, but exhibited decreased branch number in *Arabidopsis*^49,50^. Silencing of *GhFT* in domesticated and day-neutral cotton resulted in more vegetative branches and delayed flowering time^21^, which are similar to the phenotypes of *GhHB12*-overexpression line. As expected, up-regulation of *GhrSPL10* partially rescued the phenotypes of bushy architecture and delayed flowering of *GhHB12*-overexpressing plants (Fig. 3); the expression of *GhFT*, *GhFUL*, and *GhAP1* was supressed in *GhHB12*-overexpressing cotton plants, while overexpression of *GhrSPL10* partially attenuated this suppression (Fig. 4a). Furthermore, transient expression of *GhFT* in *GhHB12*-overexpressing plants also partially rescued the phenotypes of bushy architecture and delayed flowering (Fig. 4b–e). Unexpectedly, GhrSPL10 could up-regulate *GhSOC1* and *GhAP1*, but not *GhFT* and *GhFUL* (Fig. 4a and Supplementary Figure 5a), presumably because *GhSOC1* was the direct target of GhrSPL10, while *GhFT* and *GhFUL* were the direct targets of GhHB12, and *GhAP1* was the direct target of both GhrSPL10 and GhHB12. When *GhHB12* and *GhrSPL10* were co-overexpressed in cotton, they inhibited each other and subsequently regulated the expression of *GhSOC1*, *GhFT*, *GhFUL*, and *GhAP1* antagonistically. Additionally, the variations in the expression of *GhSPL2* and *GhSPL3* under the regulation of GhHB12, GhrSPL10, and GhmiR157 were similar to those of *GhFT* and *GhFUL* (Supplementary Figure 5a). All results indicated that there is a complex regulatory network composed of GhHB12, GhmiR157-GhSPLs, and GhFT/GhSOC1/GhFUL/GhAP1 that regulates the photoperiodic flowering of cotton during domestication. Therefore, further research is needed to clarify which of them were selected for during cotton domestication and how the genes related to cotton flowering time are regulated by the interactions between GhHB12 and GhSPLs or other proteins.

Previous studies have shown that *GhFT* is regulated by daily oscillations in photoperiod and contributes to cotton photoperiod sensitivity^21,51^. We found that both *GhHB12* and *GhFT* are regulated by the circadian clock and photoperiod (Supplementary Figure 5c), and the GhHB12-mediated phenotypes were rescued under short-day conditions (Supplementary Figure 5d–f). It is consistent with the enrichment of light responsive cis-acting elements in the promoter of *GhHB12* (Supplementary Figure 1d). Additionally, in our previous study, the expression of *GhHB12* was elevated by ABA and salt treatment^31^. Most HD-Zip I class transcription factors were reported to be involved in developmental reprogramming in response to changes of environmental conditions^52,53^ (e.g. drought, salt, and light). Although the molecular basis underlying the rescue of the GhHB12-mediated phenotypes under short-day conditions has not been clarified in this report, our results suggested that GhHB12 is a node of convergence between cotton development and response to various environmental changes. Hence, further investigation of the possible post-translational modification and protein modification of GhHB12 will expand our understanding of the functions of GhHB12 in cotton growth and development under different conditions.

In conclusion, the functions of the GhHB12 and GhMir157-GhSPLs pathways revealed in this study have significant implications for understanding the regulation of cotton architecture and phase transition, as well as for the breeding of early mature cotton varieties suitable for mechanical harvest.

## Methods

### Plant materials and growth conditions

Domesticated upland cotton (*Gossypium hirsutum*) YZ1 and TM-1 and ancestral upland cotton *latifolium*, *palmeri*, *punctatum*, *yucatanense*, *marie-galante*, *morilli*, and *richmonolii* were used in the experiments. The wild-type (YZ1), transgenic cotton plants, and ancestral upland cotton were grown under natural long-day conditions in the experimental field of Huazhong Agricultural University (Wuhan, China, 30.4°N, 114.2°E) during summer and were grown under natural short-day conditions in the field of Sanya (Hainan, China, 18.2°N, 109.5°E) during winter with normal daily management.

### Construct generation and transformation

The cDNA of *GhHB12* was cloned into the pGWB418 plasmid; a genomic sequence containing *GhmiR157* precursor and *MIMI157* (mimicry *GhmiR157*) was cloned into the pGWB402 plasmid^33,34^. GhrSPL10 was generated by two rounds of mutagenic PCR and then cloned into the pGWB408 plasmid to produce overexpression plants^35^. The vector pHellesgate4 was used for *GhHB12*-RNAi. A 969-bp promoter sequence of *GhHB12* was cloned into the vector pGWB433. Agrobacterium-mediated transformation was carried out to generate transgenic cotton and tomato plants, and YZ1 (cotton) and A57 (tomato) were used as wild-type and transgenic receptors.

### GUS (β-glucuronidase) staining

*proGhHB12:GUS* transgenic *Arabidopsis* seedlings were incubated in GUS staining solution for 12 h at 37 °C and then washed with 75% ethanol. The GUS staining solution was composed of 0.9 g l^–1^ 5-bromo-4-chloro-3-indolylglucuronide, 50 mM sodium phosphate buffer (pH 7.0), 20% (v/v) methanol, and 100 mg l^–1^ chloromycetin. The samples were examined and photographed with a stereomicroscope (Leica Microsystems, Germany).

### Southern blotting and qRT-PCR analysis

Genomic DNA was extracted from the young leaves of wild-type (YZ1) and transgenic cotton plants using a DP305 plant genomic DNA kit (Tiangen Biotech, Beijing), digested with *Hind III* and resolved in 0.8% agarose gel, then transferred to Hybond N^+^ nylon membranes (Amersham, UK). Fragment of *NPTII* was used as probes and labeled with 32P (Promega Labeling Kit, Promega). Hybridization was performed at 65 °C for 16–18 h and was followed by washing two times in 2 × SSC, for 5 min each, and then two times in 0.1 × SSC, for 10 min each, to remove unbound probes. Then the washed membranes were scanned using FLA-5000 Plate/Fluorescent Image Analyzer (Fuji Poto Film, Tokyo, Japan). qRT-PCR was performed using the 7500 Real-Time PCR System (ABI, Foster City, USA) with SYBR Green (Bio-Rad, USA). The *GhUBQ7* (GenBank: DQ116441) gene from *G. hirsutum* was used as the endogenous reference gene. The relative transcript level was determined and normalized using the reference level and averaged over the three technical replicates. The primers used in this study are listed in Supplementary Data 1. The raw gel image of the RT-PCR results is shown in Supplementary Figure 6.

### Yeast two-hybrid assays

The full-length sequences were cloned into pGADT7. pGBKT7 fused with *GhHB12* was used to transform Y2H, and pGADT7 fused with *GhSPL2*, *GhSPL3*, *GhSPL5, GhSPL9*, *GhSPL10*, and *GhSPL13* was transferred into Y187 yeast using the Transformation System (Clontech, Cat. no. 630489). Interactions between these transcription factors after mating were determined by growth on SD medium with the -Ade/-His/-Trp/-Leu assay as described by the manual (Clontech, Cat. no. 630489).

### Virus inoculations

dCLCrV a disarmed geminivirus *Cotton leaf crumple virus* was used for virus-induced flowering^21^. CLCrVB, dCLCrV, and dCLCrV: GhFT vectors were generated previously^21^, and were introduced into *A. tumefaciens* strain GV3101. *A. tumefaciens* containing CLCrVB and *A. tumefaciens* containing dCLCrV or dCLCrV: GhFT were mixed in equal amounts and infiltrated into the cotyledons of *GhHB12*-overexpressing seedlings at 5 days post germination by syringe infiltration. Inoculated seedlings were covered overnight at 25 °C, and maintained in a growth chamber at 25 °C under long-day conditions (16 h light/8 h dark) for 3 weeks, and then were transplanted into the glasshouse at 26–30 °C for long-day conditions (14 h light/10 h dark).

## Supplementary Information

Supplementary Information

Description of Additional Supplementary Files

Supplementary Data 1

Supplementary Data 2

## Data Availability

The authors declare that all the data generated during this study are available in the manuscript, figures and Supplementary Information Files. Source data underlying the graphs and charts presented in the figures are available in Supplementary Data 2.
