# Supplementary material for: The cotton HD-Zip transcription factor GhHB12 regulates flowering time and plant architecture via the GhmiR157-GhSPL pathway
Source: Commun Biol. 2018 Dec 13;1:229. doi: 10.1038/s42003-018-0234-0 (PMC6292863; doi:10.1038/s42003-018-0234-0)
Supplement: Supplementary file 1 — Supplementary Information [file 42003_2018_234_MOESM1_ESM.pdf]

Supplementary Figure

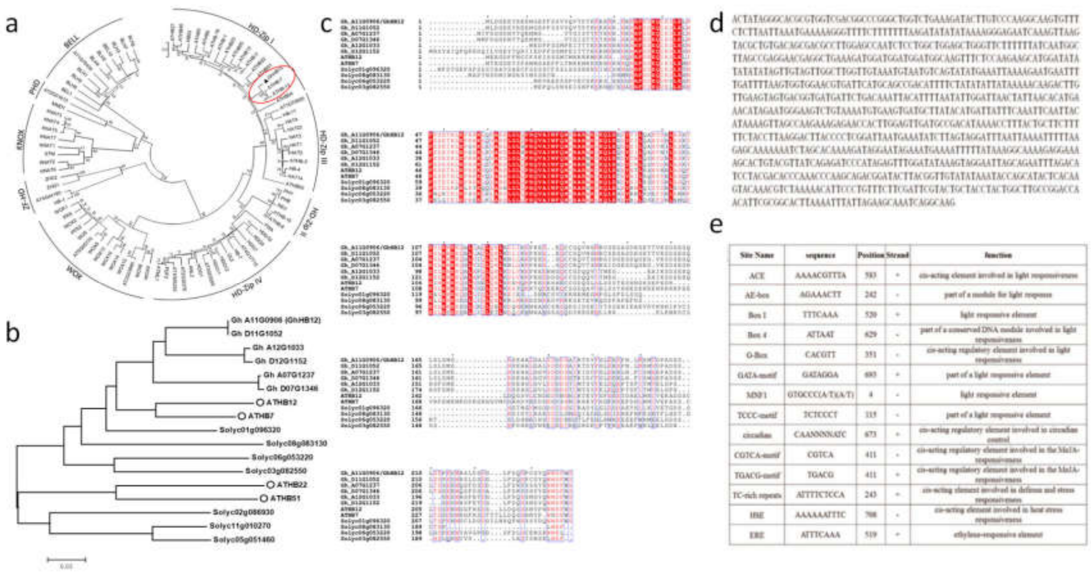

Supplementary Figure 1. Alignment and phylogenetic analysis of GhHB12 and homeodomain superfamily proteins from *Arabidopsis*. a, Phylogenetic analysis of GhHB12 and homeodomain superfamily proteins from *Arabidopsis*. **b**, Phylogenetic analysis of GhHB12 homologs proteins from cotton, *Arabidopsis* and tomato. **c**, Alignment of GhHB12 homologs proteins from cotton, tomato and *Arabidopsis* HD-ZIP I subfamily proteins ATHB7 (NP_182191.1) and ATHB12 (NP_191748.1). **d**, The sequence of the *GhHB12* promoter (-981 bp to +15 bp). **e**, The *cis*-acting elements involved in environment responsiveness in the GhHB12 promoter.

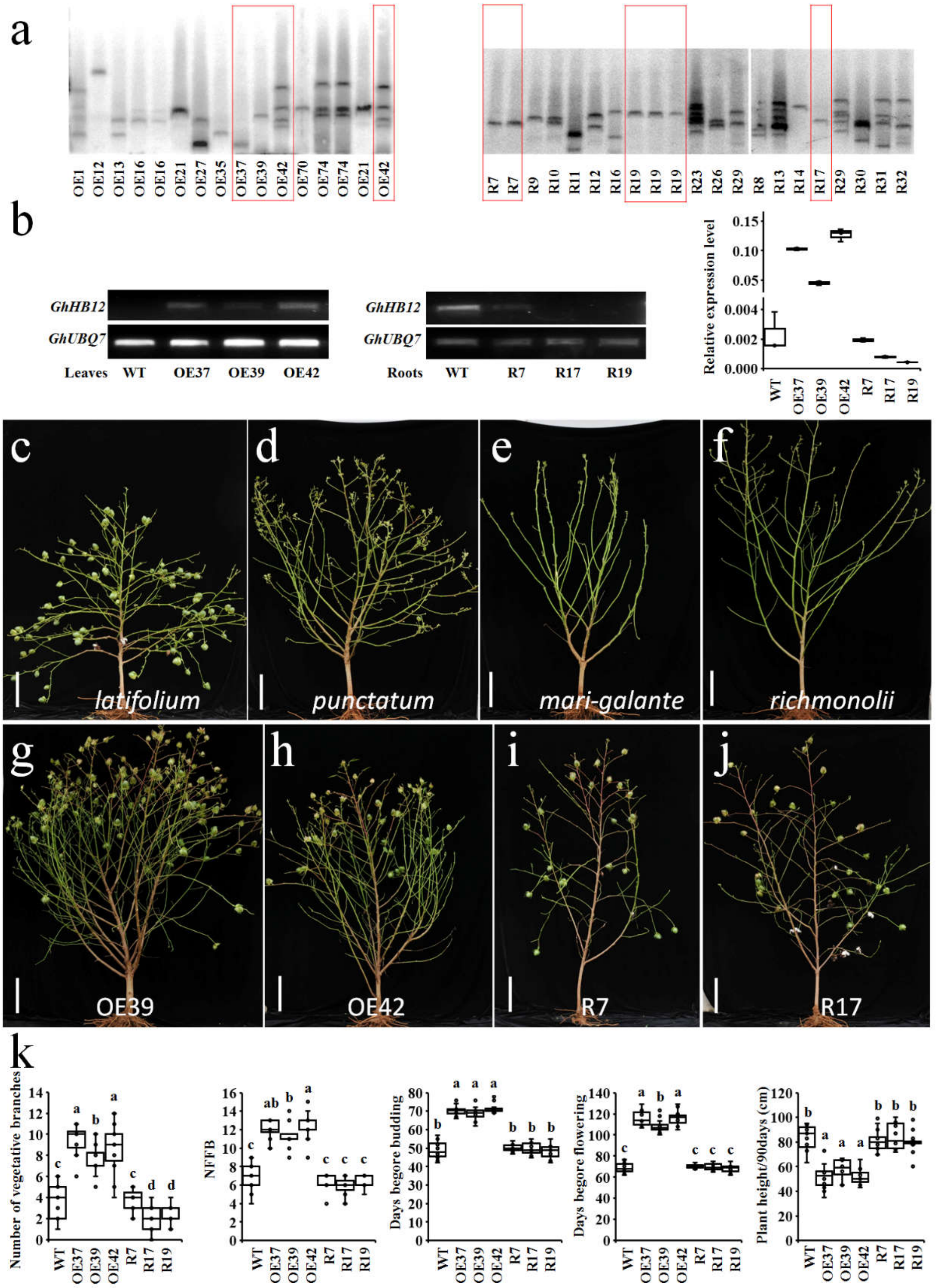

Supplementary Figure 2. Regulation of GhHB12 on cotton vegetative branches and reproductive development. a-b, Southern blotting (**a**), expression level analysis by RT-PCR and qRT-PCR (**b**) of *GhHB12*-overexpressing and RNAi cotton plants. *NPTII* was used as the Southern blotting probe, λ NDA digested with *HindIII* was used as the marker, and *GhUBQ7* was used as the reference gene. The data represent

the mean ± SD of three technical replicates. **c-j**, Photographs of ancestral upland cotton *latifolium* (**c**), *punctatum* (**d**), *marie-galante* (**e**) and *richmonolii* (**f**), *GhHB12*-overexpressing in YZ1 background OE39 (**g**) and OE42 (**h**), *GhHB12* RNAi in YZ1 background R7 (**i**) and R17 (**j**) plants. Scale bars, 20 cm. **k**, Comparison of the number of vegetative branches, NFFB, days before budding and flowering, plant height (90 days) among YZ1, *GhHB12*-overexpressing, and *GhHB12* RNAi plants. *Error bars* indicate the standard deviation of 12-15 plants, and different letters indicate significant differences at $P < 0.05$ (Duncan's multiple range test).

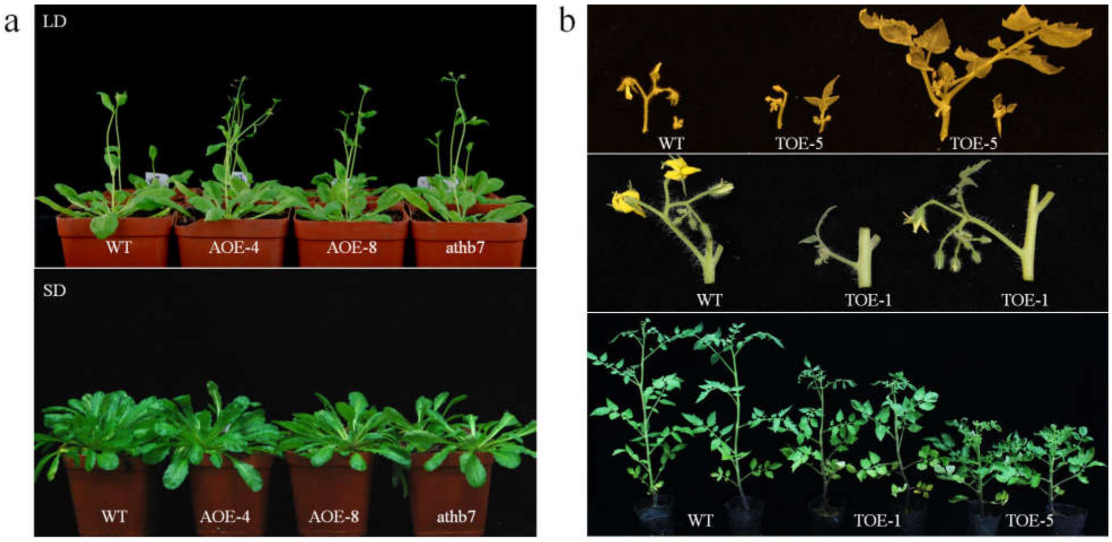

Supplementary Figure 3. Identification of GhHB12 in *Arabidopsis* and tomato. a, Photographs of *GhHB12*-overexpressing (AOE-4 and AOE-8), athb7 and wild-type *Arabidopsis* plants in long-day (LD) and short-day (SD) conditions. **b**, Photographs of *GhHB12*-overexpressing (TOE-1 and AOE-5) and wild-type tomato (A57) plants under long-day conditions.

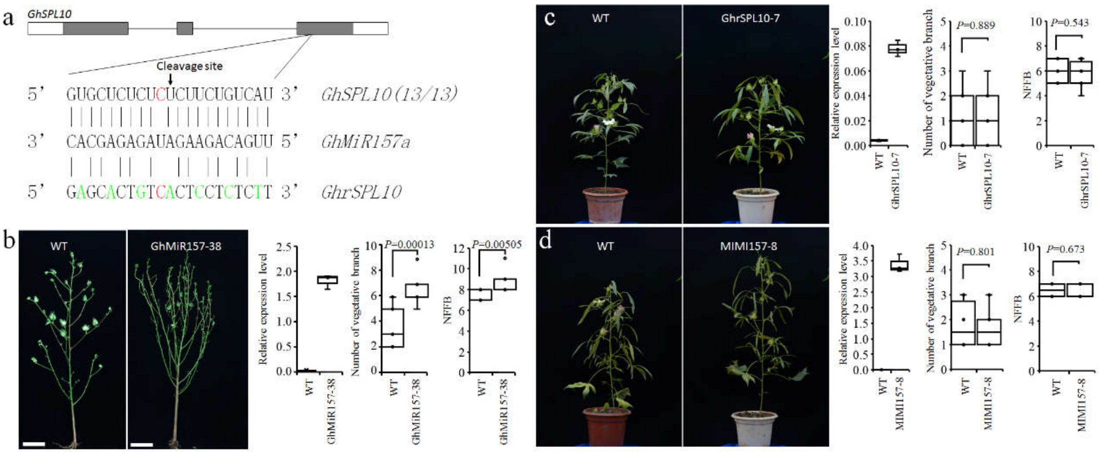

Supplementary Figure 4. Identification of *GhmiR157* and *GhSPL10*. a, RLM-RACE analysis of the *GhmiR157a* cleavage sites in YZ1. Black arrows indicate the position of the target cleavage sites. The numbers beside black arrows indicate the cleavage frequency. *GhrSPL10*, a *GhmiR157*-resistant version without the *GhmiR157* response element. **b-d**, Comparison of the number of vegetative branches and NFFBs between WT and GhmiR157-38 (**b**) plants, WT and GhrSPL10-7 (**c**) plants, WT and MIMI157-8 (**d**) plants. A *p < 0.05 was indicate significant differences between transgenic and WT (wild-type) (Student's t-test). GhmiR157-38, *GhmiR157*-overexpression line 38; GhrSPL10-7, *GhrSPL10*-overexpression line 7; MIMI157-8, mimicry *GhmiR157*-overexpression line 8.

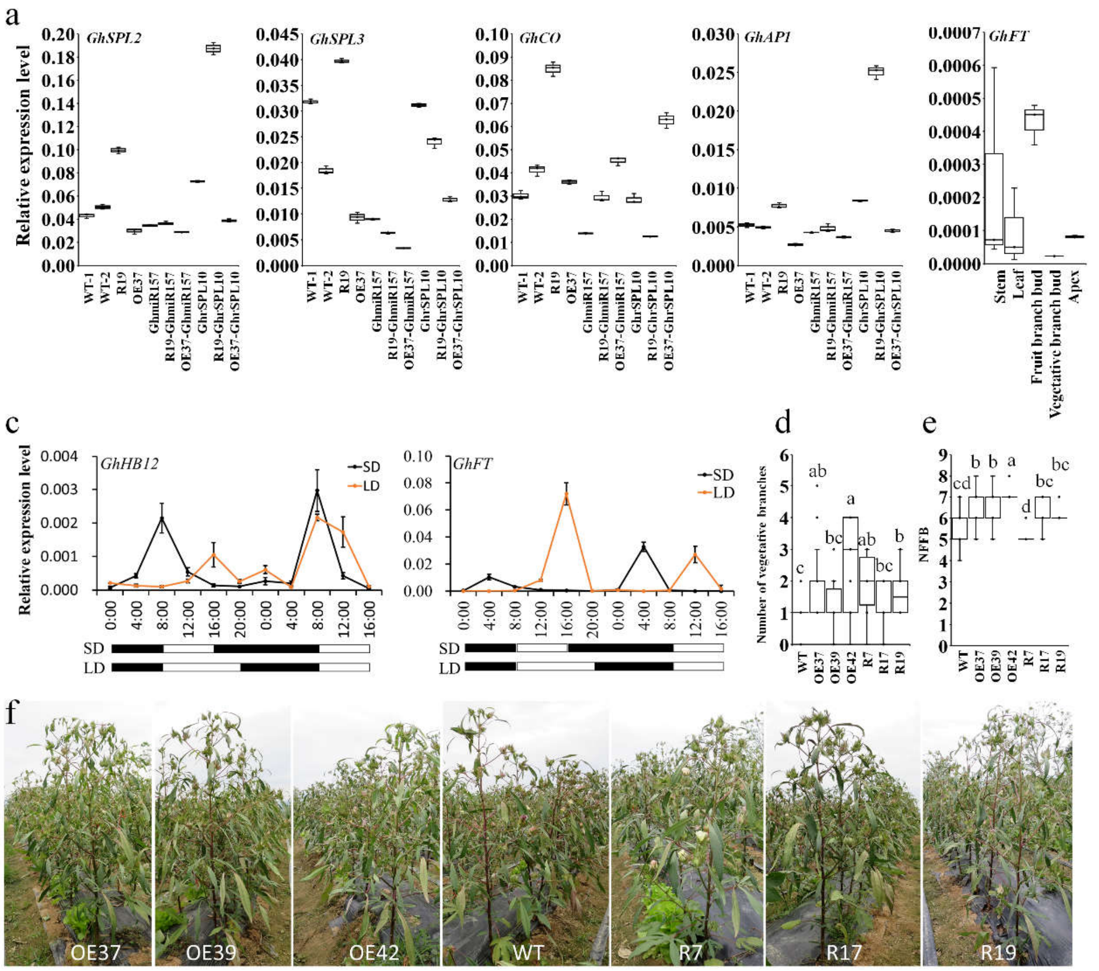

Supplementary Figure 5. Regulation of GhHB12 on cotton photoperiod sensitivity. a, Expression analysis of the cotton flowering time genes in *GhHB12*, *GhmiR157*, and *GhrSPL10* transgenic cotton seedlings. **b**, Detection of the expression levels of *GhFT* in the stem, leaf, fruit branch bud, vegetative branch bud, and apex of domesticated upland cotton (*Gossypium hirsutum* L. YZ1) by qRT-PCR. **c**, qRT-PCR analysis of *GhHB12* and *GhFT* in YZ1 seedlings under long-day (LD: 16 h light/8 h dark) or short-day (SD: 8 h light/16 h dark) conditions. The open and filled bars at the bottom represent the light and dark periods, respectively. The *GhUBQ7* gene was used as the endogenous reference gene. The data represent the mean ± SD of three technical replicates. **d-f**, Photographs (**f**), number of vegetative branches (**d**) and NFFB (**e**) of WT and *GhHB12*-transgenic cotton plants grown in the Hainan Island winter nursery (natural short-day). Error bars indicate the standard deviation of 10-22 plants, and different letters indicate significant differences at $P < 0.05$ (Duncan's multiple range test).

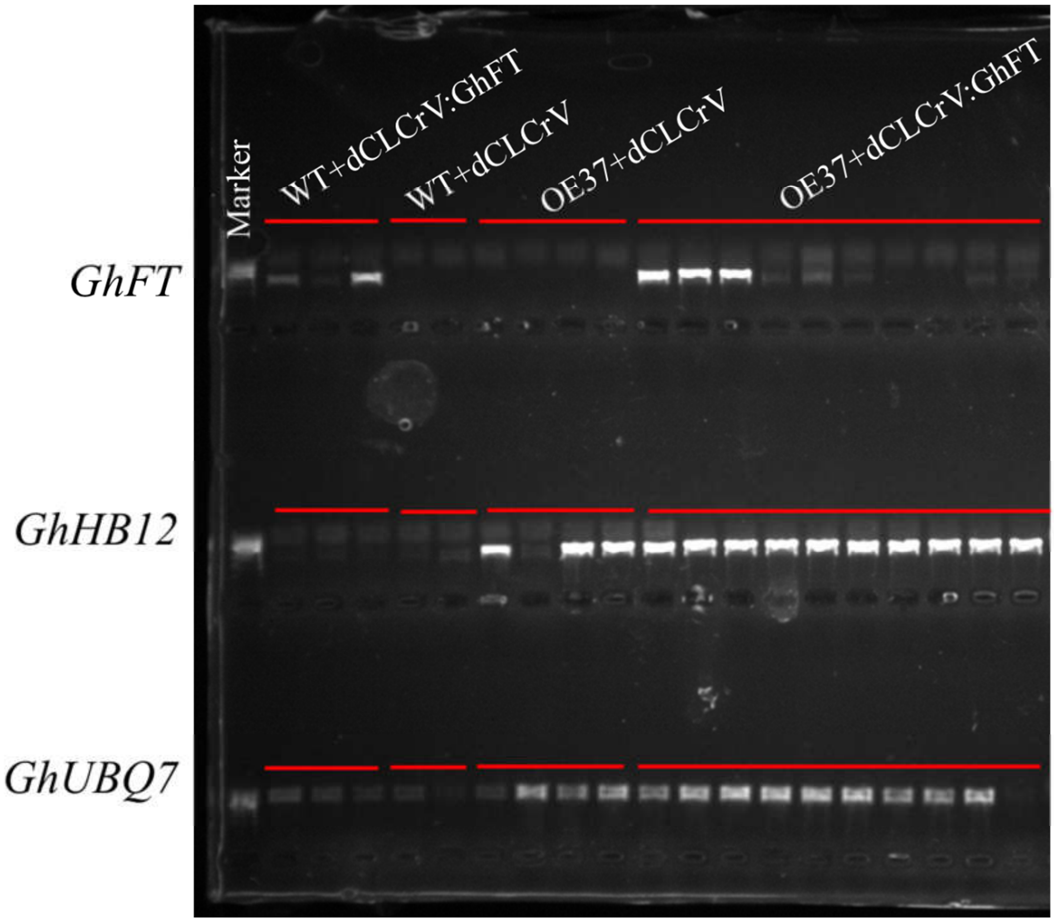

Supplementary Figure 6. RT-PCR analysis of the transcript levels of *GhHB12* and *GhFT* in WT and GhHB12-overexpressing plants inoculated with dCLCrV or dCLCrV:GhFT.
