# Supplementary material for: The cotton HD-Zip transcription factor GhHB12 regulates flowering time and plant architecture via the GhmiR157-GhSPL pathway
Source: Commun Biol. 2018 Dec 13;1:229. doi: 10.1038/s42003-018-0234-0 (PMC6292863; doi:10.1038/s42003-018-0234-0)
Supplement: Supplementary file 2 — Description of Additional Supplementary Files [file 42003_2018_234_MOESM2_ESM.docx]

Descriptions of Additional Supplementary Files

**File Name:** Supplementary Data 1

**Description:** Primers used in this study. Primer Names: a pair of primers includes -S (Sense primer) and –A (Anti-sense primer); Sequences (5'-3'): the sequences of adapter were shown as bold fonts; Gene ID: the target genes of the primers.

**File Name:** Supplementary Data 2

**Description:** The source data underlying the graphs and charts presented in figures. The source data (Relative gene expressions, Number of vegetative branches, NFFB, Days before budding, Days before flowering and Height of 90-day plants) underlying the graphs and charts presented in Figure 1-4 and Supplementary figure 2,4,5.
